# Discovery of novel plasma biomarker ratios to discriminate traumatic brain injury

**DOI:** 10.12688/f1000research.20445.1

**Published:** 2019-09-27

**Authors:** Michelle Chen, Antoninus Soosaipillai, Douglas D. Fraser, Eleftherios P. Diamandis

**Affiliations:** 1 Department of Laboratory Medicine and Pathobiology, University of Toronto, Toronto, Ontario, Canada; 2 Department of Pathology and Laboratory Medicine, Mount Sinai Hospital, Toronto, Ontario, Canada; 3 Department of Pediatrics, Western University, London, Ontario, Canada; 4 Department of Clinical Biochemistry, University Health Network, Toronto, Ontario, Canada; 5 Lunenfeld-Tanenbaum Research Institute, Mount Sinai Hospital, Toronto, Ontario, Canada

**Keywords:** Kallikrein 6, prostaglandin D synthase, concussion, biomarker, biomarker ratios, traumatic brain injury

## Abstract

**Background: **Traumatic brain injury (TBI) is a major cause of death and disability. Despite increased awareness, reliable biomarkers are urgently needed to aid in all forms of traumatic brain injury diagnosis and prognosis.

**Methods: **Here, we aim to assess the diagnostic utility of known and novel TBI biomarkers in a pilot patient cohort of severe TBI (sTBI) patients and healthy controls. We analyzed concentrations of S100 calcium binding protein B (S100B), neuron specific enolase (NSE), human kallikrein 6 (hK6) and prostaglandin D2 synthase (PGDS) using ELISA immunoassays.

**Results: **Plasma levels of hK6 and PGDS were significantly lower in sTBI compared with controls, while S100B and NSE were significantly higher. Furthermore, we show that ratios of NSE and S100B with hK6 and PGDS may be able to determine the presence of sTBI better than single markers alone.

**Conclusions: **The findings presented here represent a starting point for future validation, where biomarker ratios can be tested in independent TBI cohorts.

## Abbreviations

TBI: Traumatic brain injury, mTBI: mild traumatic brain injury, sTBI: severe traumatic brain injury, S100B: S100 calcium binding protein B, NSE: neuron specific enolase, hK6: human kallikrein 6, PGDS: prostaglandin D2 synthase, GCS: Glasgow Coma Scale, CT: computed tomography, UCH-L1: ubiquitin C-terminal hydrolase-L1, GFAP: glial fibrillary acidic protein, PPV: positive predictive value, ROC: receiver operating characteristic, AUC: area under the curve, CI: confidence interval, LOS: length of stay

## Introduction

Traumatic brain injury (TBI) is a significant cause of morbidity, with an annual incidence of over 65 million cases worldwide ^1^. TBI is caused by trauma to the head or body that causes neurologic damage and dysfunction. It occurs most often due to falls, but is also a common occurrence in motor vehicle collisions, sports and assaults ^2^. These mechanical injuries can vary in both the severity and the form of injury to the brain ^3, 4^. Generally, presenting TBI may be classified as mild, moderate, or severe. Patients are primarily stratified using the Glasgow Coma Scale (GCS), a scoring system that evaluates verbal performance, motor function and eye function. A lower GCS score indicates a more severe diagnosis and may therefore indicate the need for further investigations and possibly medical and/or surgical interventions.

Given the significant burden of disease, reliable diagnostic and prognostic approaches are urgently needed to guide care. One promising solution is the quantification of blood biomarkers capable of delineating the severity of trauma and/or prognostic outcome. These proteins enter the circulation due to disruption of brain structures such as the blood brain barrier and/or axons, leading to a neuroinflammatory response ^5, 6^. Although various markers of mTBI have been identified, a validated panel has yet to be resolved ^7^. For example, S100 calcium-binding protein B (S100B) and neuron-specific enolase (NSE) are two well-documented biomarkers with good correlations to TBI outcome. However, their performance may be limited by non-specific correlations ^8, 9^. The Food and Drug Administration recently approved the first blood biomarker test to guide head imaging after TBI; it measures ubiquitin C-terminal hydrolase-L1 (UCH-L1) and glial fibrillary acidic protein (GFAP) ^10^. Despite the approval, concerns have been raised regarding the relatively low positive predictive value (PPV) of the test and its utility as a marker for TBI diagnosis specifically ^11^. Given this, it is clear that there is a need for improved diagnostic TBI biomarkers with validated efficacy in a clinical setting.

Human kallikrein 6 (hK6) is a serine protease with high brain and spinal cord expression, particularly in oligodendrocytes ^12, 13^. Previous studies have linked hK6 to brain-related disorders such as spinal cord injury, where it may play a role in myelin repair ^14, 15^. Furthermore, work by our group has demonstrated that serum hK6 may have significant clinical utility as a prognostic marker of non-traumatic subarachnoid hemorrhage ^16^. Prostaglandin D synthase (PGDS) is a highly expressed enzyme that represents nearly 3% of the total protein in cerebral spinal fluid ^17^. The role of PGDS in hypoxic-ischemic brain injury has been well-characterized ^18, 19^. Therefore, we hypothesize that hK6 and PGDS may be novel biomarkers of TBI and may have predictive value in its diagnosis.

In this study, we measured plasma concentrations of multiple proteins in sTBI patients compared with healthy controls. These markers include those well established in literature (S100B and NSE), as well as those we hypothesize to be detectable in plasma following TBI – hK6 and PGDS. We reason that the concentration of these proteins will fluctuate following brain injury, causing them to be more or less abundant in TBI patient plasma compared with controls. Furthermore, we hypothesized that the combination of these proteins may be useful biomarkers to delineate the degree of TBI in patient populations.

## Methods

### Study population and sample collection

Plasma samples were obtained using strict standard operating procedures from the Translational Research Centre, London, Ontario, Canada ( https://translationalresearchcentre.com/). Study protocols were approved by The Western University Health Science Research Ethics Board (REB # 16693) and by The Mount Sinai Hospital Research Ethics Board (REB #18-0069-E). Informed consent was obtained either from the patient or a substitute decision maker.

Blood samples were drawn from adults with severe TBI (sTBI; n=10), as defined by GCS ≤ 8 with abnormal CT findings, within 24 h of hospital admission. Samples from age-/sex-matched healthy controls (n=10) were also obtained from the Translational Research Centre. Blood was collected into 3%, 0.109 M sodium citrate tubes, centrifuged for 15 min (1500 x g at 4 °C), and the plasma was frozen at −70 °C. Detailed collection and handling protocols have been described previously ^20, 21^. Plasma samples were transferred on dry ice to Mount Sinai Hospital, Toronto, Ontario, Canada and stored at -80 °C until analysis.

### Measurement of S100B and NSE

The concentrations of S100B and NSE were measured using electrochemoluminometric immunoassay (Cobas e411 Immunoanalyzer; Roche Diagnostics, Penzberg, Germany) according to the manufacturer’s protocol, with a detection limit of 0.02 ng/mL and 0.19 ng/mL, respectively. All samples were thawed at room temperature and analyzed in one run.

### Measurement of hK6 and PGDS

For all hK6 measurements, samples were thawed at room temperature, then diluted 10 fold with 6% BSA (Wisent Bioproducts, Quebec, Canada). hK6 was analyzed with an in-house spectrophotometric ELISA assay with a detection limit of 0.078 ng/mL and dynamic range of 0.078–5 ng/mL ^22^. Briefly, samples were analyzed in duplicate using an hK6 monoclonal capture antibody and mouse monoclonal biotinylated detection antibody. White polystyrene 96-well plates were coated with 400 ng of capture antibody in 100 μl of coating buffer (50 mmol/L Tris-HCl) and incubated overnight. Plates were then washed in wash buffer (10 mmol/L Tris-HCl buffer, 150 mmol/L NaCl and 0.5 mL/L Tween 20). 50 μl of diluted sample and 50 μl of green assay buffer (60 g/L BSA, 0.1 g/L goat globulin, 0.02 g/L mouse globulin, 1 g/L bovine globulin, 5 ml/L Tween 20 and 37 g/L KCl) were added to each well and incubated with shaking for 2 h. Plates were washed, incubated with 50 ng (100 μl) of detection antibody for 1 h, washed, and incubated again with 5 ng (100 μl) of alkaline phosphatase-conjugated streptavidin for 15 min. 100 μl of diflunisal phosphate solution (0.1 M NaOH containing 10 mM diflunisal phosphate) diluted 1:20 in substrate buffer (0.1 M Tris, 0.15 M NaCl, 1 mM MgCl2 and 0.05% NaN3) was added to each well and incubated for 10 min. The reaction was stopped with 100 µl/well of developing solution (1 M Tris, 0.15 M NaOH, 2 mM TbCl3 and 3 mM EDTA). Excitation at 340 nm and emission at 615 nm was measured with a time-resolved fluorometer (EnVision, Perkin-Elmer). Samples were diluted 200 fold in 6% BSA for PGDS analysis via similar in-house ELISA, with a detection limit of 0.2 ng/mL and dynamic range of 0.2–50 ng/mL, as described in detail elsewhere ^23^.

### Data analysis

Ratios of NSE/hK6, S100B/hK6, NSE/PGDS, S100B/PGDS were calculated for data analysis. Statistical analyses were conducted using GraphPad Prism version 6.0e. For receiver operating characteristic (ROC) analysis, the area under the curve (AUC) estimates with 95% confidence intervals (CIs) were calculated using DeLong’s method ^24^. CIs were calculated using 2000 bootstrap replicates each. P-values were calculated using Wilcoxon signed-rank tests. Spearman correlations were conducted between biomarker value and lowest-documented GCS score (within 24 h of hospital admission), as well as hospital length of stay (LOS). Mann-Whitney U test was used to compare biomarker distribution in patients vs. controls. Two-tailed student’s t-test was used to compare biomarker concentrations between patient and control groups. One sTBI subject with extreme high values for all assays was omitted from data analysis due to potential sample contamination or processing error.

## Results

### Clinical characteristics of recruited patients

Demographics and pathological characteristics of recruited patients are summarized in Table 1. All raw clinical and biomarker data are available as *Underlying data* ^25^.

**Table 1.** Demographic and clinical information of study participants.

Variable	sTBI (n=10)	Healthy controls (n=10)
Age, years *	35.1 (14.4)	35.5 (14.3)
Sex, male/female	7/3	7/3
Lowest documented GCS * ^†^	5.1 (1.45)	
Outcome, dead/alive	4/6	
ICU LOS, days *	9.1 (6.7)	
Hospital LOS, days *	15.4 (18.0)	
Etiology, n (%)		
*Motor vehicle collision*	6 (60)	
*Falls*	3 (30)	
*Abuse/assaults (non accidental)*	1 (10)	
Admission CT abnormalities, n (%)		
*Subarachnoid hemorrhage*	5 (50)	
*Subdural hemorrhage*	4 (40)	
*Epidural hemorrhage*	2 (20)	
*Intraventricular hemorrhage*	3 (30)	
*Intraparenchymal hemorrhage*	5 (50)	
*Diffuse axonal injury*	6 (60)	
*Midline shift*	2 (20)	
*Tonsillar herniation*	1 (10)	
*Cerebral contusion*	8 (80)	
*Cerebral edema*	2 (20)	
*Basal skull fractures*	6 (60)	
*Hydrocephalus*	1 (10)	
*Pneumocephalus*	2 (20)	

GCS, Glasgow Coma Score; ICU, intensive care unit; LOS, length of stay. *Median (SD). ^†^Within 24 h of hospital admission.

### Use of single biomarkers and ratios to predict sTBI

**Figure 1.**
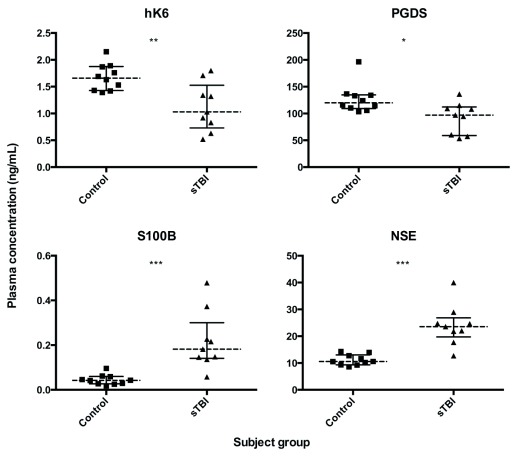
Individual biomarker distribution in the plasma of sTBI patients (n=9) and healthy controls (n=10). Dotted lines represent group median, bars show interquartile range. Mann Whitney U test; * *p* < 0.05, ** *p* < 0.01, *** *p* < 0.001, *ns* not significant.

**Figure 2.**
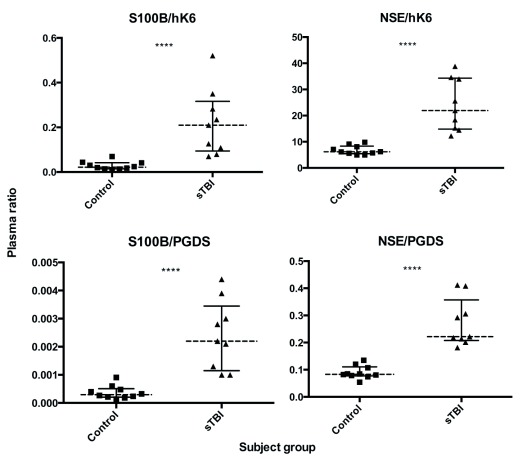
Plasma biomarker ratios in sTBI patients (n=9) and healthy controls (n=10). Dotted lines represent group median, bars show interquartile range. Mann Whitney U test; **** *p* < 0.0001.

We used in-house ELISAs and electrochemoluminometric assays to measure biomarker concentrations in the plasma of adult sTBI patients and matched healthy controls. Plasma levels of hK6 and PGDS were significantly lower ( *p* = 0.0076 and *p* = 0.0172, respectively) in sTBI compared with controls, while S100B and NSE were significantly higher ( *p* = 0.0002 and *p* = 0.0002, respectively) ( Figure 1). All analyzed biomarker ratios (NSE/hK6, S100B/hK6, NSE/PGDS, S100B/PGDS) showed significant differences in sTBI vs control ( *p* < 0.0001, for all; Figure 2).

**Figure 3.**
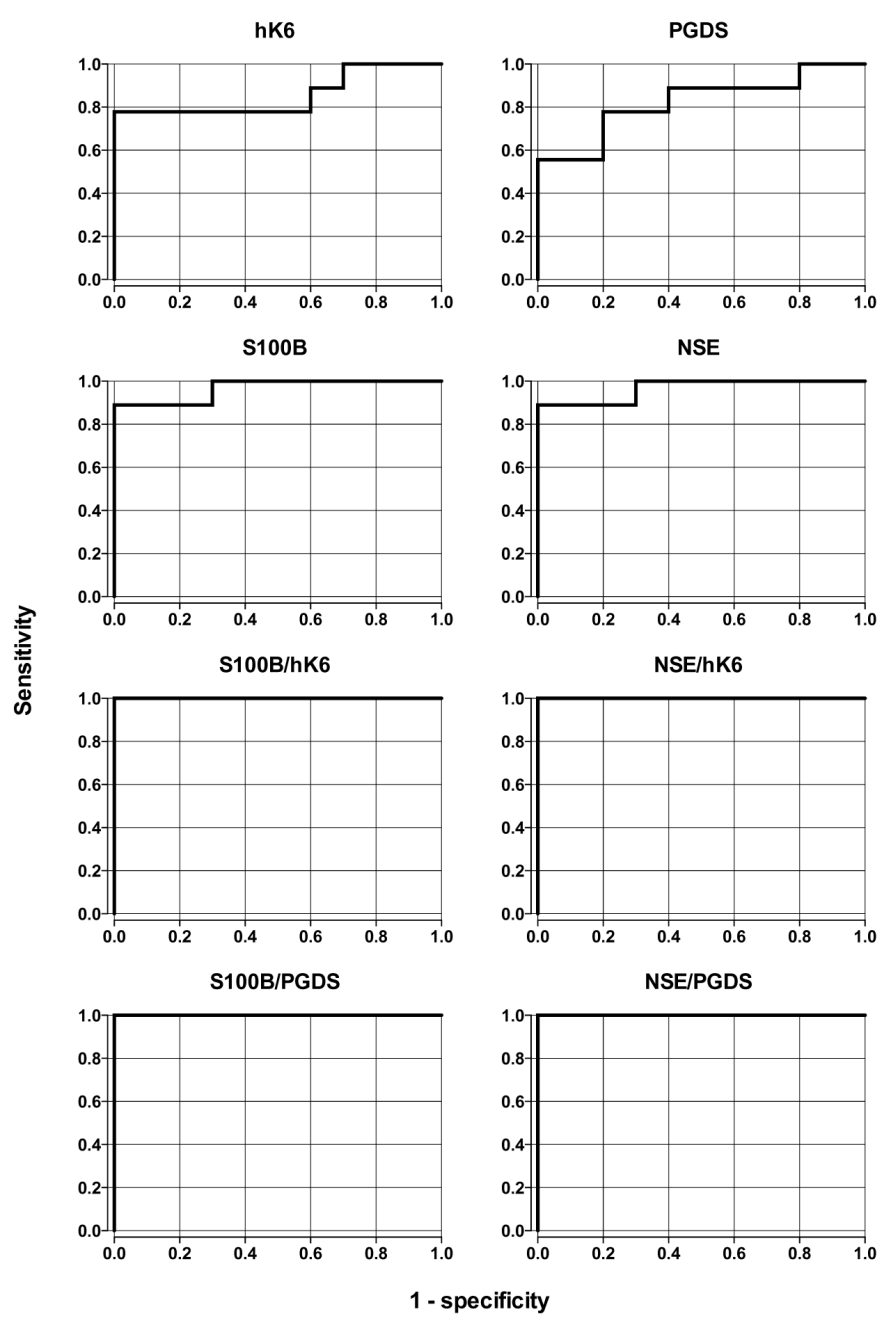
Receiver operating characteristic (ROC) curves for plasma biomarkers and biomarker ratios for sTBI diagnosis.

Individual biomarkers and calculated ratios were evaluated to determine their ability to discriminate between sTBI and control groups. AUC estimates and CIs are presented in Table 2. Plasma concentrations of NSE, S100B, hK6, PGDS and all ratios had significant predictive ability to discriminate sTBI from healthy controls ( Figure 3, Table 2). Biomarker ratios showed the highest AUC values (AUC = 1.0, for all), while NSE and S100B (AUC = 0.967 for both) performed better than hK6 (AUC = 0.856) and PGDS (AUC = 0.822).

### GCS and mortality

**Figure 4.**
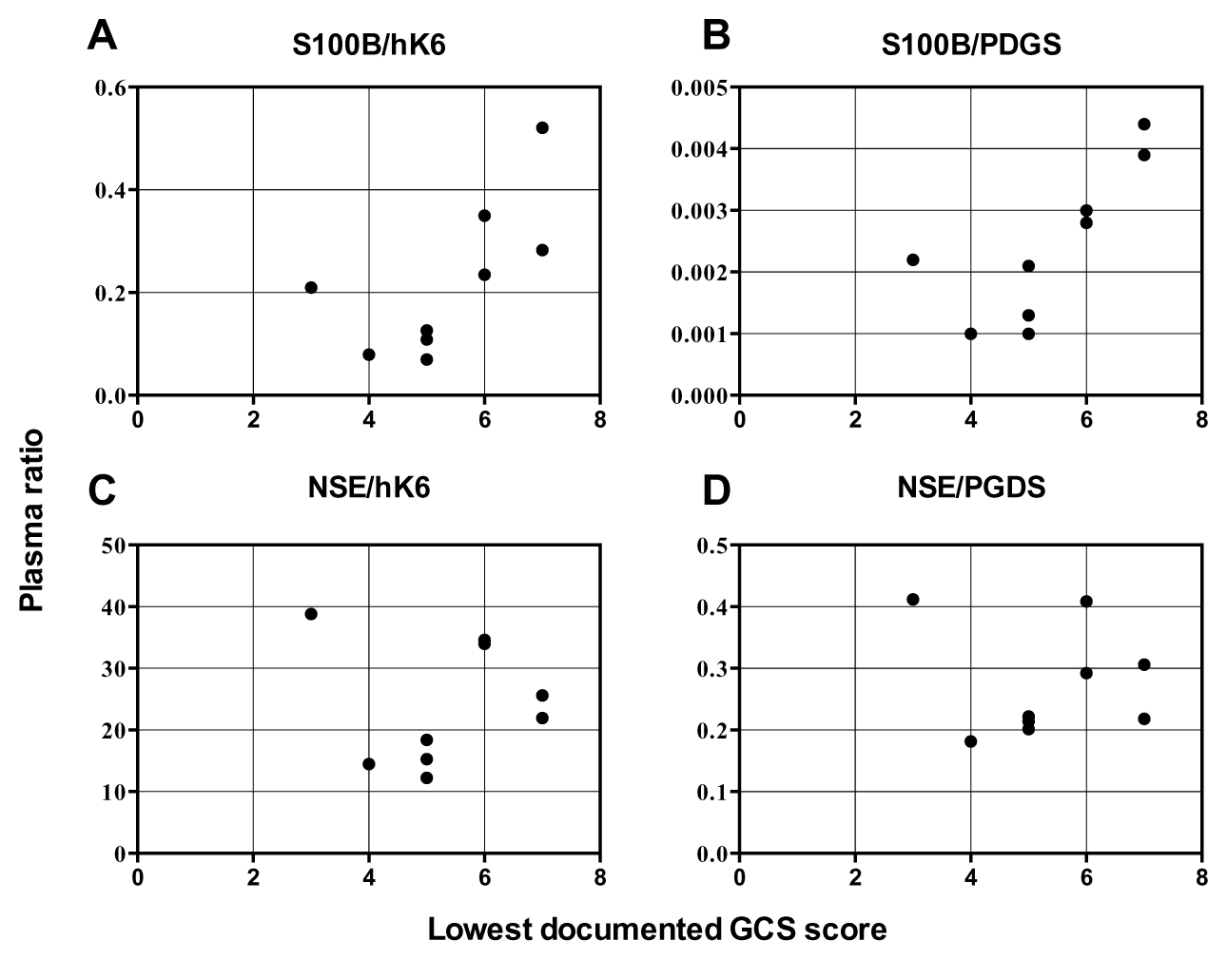
Biomarker ratio correlation with lowest documented Glasgow Coma Scale (GCS) score in patients with sTBI (n=9). Spearman’s correlation: ( **a**) S100B/hK6; *r _s_* = 0.7353, *p* = 0.0292; ( **b**) S100B/PGDS; *r _s_* = 0.7899, *p* = 0.0151; ( **c**) NSE/hK6; ns; ( **d**) NSE/PGDS; ns.

There were no significant correlations between any individual biomarker values and lowest-documented GCS score ( *Extended data*: Supplemental Figures S1, Table S1 ^26^). However, ratios of S100B/hK6 ( *r _s_* = 0.7353, *p* = 0.0292) and S100B/PGDS ( *r _s_* = 0.7899, *p* = 0.0151) were significantly associated with GCS score ( Figure 4). Finally, we did not observe any significant differences in biomarker or ratio levels between sTBI survivors and non-survivors ( *Extended data*: Supplemental Figures S2 and S3 ^26^).

**Table 2.** Single biomarker and marker ratio area under the curve (AUC) values for sTBI diagnosis.

Biomarker	AUC	95% CI	*P* value
S100B/hK6	1.0000000	(1, 1)	0.0000217
NSE/hK6	1.0000000	(1, 1)	0.0000217
S100B/PGDS	1.0000000	(1, 1)	0.0000217
NSE/PGDS	1.0000000	(1, 1)	0.0000217
NSE	0.9666667	(0.89, 1)	0.0001516
S100B	0.9666667	(0.89, 1)	0.0001516
hK6	0.8555556	(0.66, 1)	0.0076209
PGDS	0.8222222	(0.62, 1)	0.0172119

### Hospital LOS

**Figure 5.**
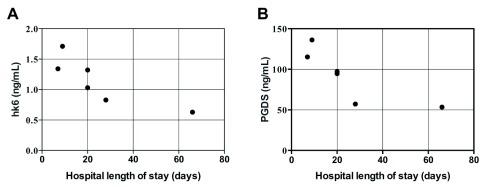
Correlation between hospital length of stay (LOS) in days and plasma biomarker abundance in surviving sTBI patients (n=6). Spearman’s correlation: ( **a**) hK6; *r _s_* = -0.9276, *p* < 0.006; ( **b**) PGDS; *r _s_* = -0.9276, *p* < 0.006.

Plasma concentrations of biomarkers and ratios were compared to hospital length of stay (LOS) of surviving sTBI patients. LOS was significantly correlated with hK6 ( *r _s_* = -0.9276, *p* < 0.006) and PGDS ( *r _s_* = -0.9276, *p* < 0.006), with lower biomarker levels corresponding to longer LOS ( Figure 5, non-significant data not shown).

## Discussion

In this pilot study, we measured plasma concentrations of multiple proteins in sTBI patients compared with age-/sex-matched healthy controls. We analyzed concentrations of S100B, NSE, kallikrein 6 (hK6) and prostaglandin D2 synthase (PGDS). We show that ratios of NSE and S100B with hK6 and PGDS may be able to determine the presence of sTBI with 100% accuracy.

Plasma levels of hK6 and PGDS were significantly lower in sTBI compared with controls, while S100B and NSE were significantly higher ( Figure 1). This is consistent with abundant research demonstrating increased S100B and NSE post-TBI ^27– 29^. PGDS has also been shown to be reduced in the CSF of pediatric patients with inflicted TBI ^30^. To our knowledge, our findings linking hK6 with sTBI have not been previously reported.

Interestingly, the ratio of NSE/PGDS was able to segregate sTBI patients from healthy controls better than any marker alone. We observed one sTBI patient with NSE and S100B values that fell within the control range, but had a NSE/PGDS and S100B/hK6 value that was clearly higher than controls ( Figure 1 and Figure 2). This suggests that these ratios may provide better sensitivity in determining the presence of sTBI. Furthermore, ROC analysis shows higher discriminatory capability in all ratios analyzed (AUC = 1.00, *p* = 0.0000217) in comparison with S100B and NSE alone (AUC = 0.967, *p* = 0.00015 for both, Figure 3). Taken together, our data suggest that the use of known TBI biomarkers, in conjunction with PGDS and hK6, may have significant diagnostic capability.

Although we did not identify any significant correlations between individual biomarker concentrations and lowest documented GCS score ( *Extended data*: Supplemental Figures S1 ^26^), we found that ratios of S100B/hK6 and S100B/PGDS were significantly correlated with TBI severity. We observe that higher ratio values are associated with lower GCS score in sTBI patients ( Figure 4). Furthermore, plasma concentrations of hK6 and PGDS were significantly and negatively associated with hospital LOS ( Figure 5). Further investigations with a larger sample size are needed to confirm the prognostic utility of these novel findings.

The data presented here represents a pilot study exploring the utility of four biomarkers and their ratios in diagnosing sTBI. Overall, it appears that novel ratios of S100B, NSE, PGDS and kallikrein 6 may have improved TBI diagnostic capability over any one of these markers alone and may additionally have prognostic utility. Given the small sample size, these results must be validated in a larger cohort of TBI patients. Results from these experiments may provide valuable insight into TBI diagnosis and prognosis.

## Data availability

### Underlying data

Harvard Dataverse: Underlying data: Discovery of novel plasma biomarker ratios to discriminate traumatic brain injury. https://doi.org/10.7910/DVN/VMR7MS ^25^.

This project contains the following underlying data:

- biomarker_data (biomarker data, including plasma concentrations of protein biomarkers and ratio values).
- clinical_data (clinical data, including demographic information and clinical presentation data for each enrolled patient).

### Extended data

Harvard Dataverse: Extended data: Discovery of novel plasma biomarker ratios to discriminate traumatic brain injury. https://doi.org/10.7910/DVN/UOOSXE ^26^.

This project contains the following extended data:

- Supplemental Figure S1. Individual biomarker correlation with lowest documented Glasgow Coma Scale (GCS) score in patients with sTBI (n=9).
- Supplemental Figure S2. Differences in individual biomarker concentrations between sTBI patient outcomes (n=9).
- Supplemental Figure S3. Differences in plasma biomarker ratios between sTBI patient outcomes (n=9).
- Supplemental Table S1. Spearman’s correlation values between biomarkers and lowest documented GCS score in sTBI patients (n=9).

Data are available under the terms of the Creative Commons Zero "No rights reserved" data waiver (CC0 1.0 Public domain dedication).
