# Supplementary material for: Discovery of novel plasma biomarker ratios to discriminate traumatic brain injury
Source: F1000Res. 2019 Sep 27;8:1695. [Version 1] doi: 10.12688/f1000research.20445.1 (PMC6979479; doi:10.12688/f1000research.20445.1)
Supplement: Peer review history

# Peer review: Reviewer response for version 1

**DOI:** 10.5256/f1000research.22476.r58587

**Published:** 2020-01-23

**Authors:** Marco Antonio Stefani

**Affiliations:** 1 Department of Neurosurgery, Federal University of Rio Grande do Sul (UFRGS), Porto Alegre, Brazil

This is an interesting study carried out by Chen *et al*., focusing on human serum biomarkers values to improve the diagnosis and predict prognosis of severe traumatic brain injury (sTBI). They have described human kallikrein 6 (hK6) and prostaglandin D2 synthase (PGDS) as potential new markers of injury, coupled with NSE and S100B.

 The methods are clear and the results are very well described. A larger sample is necessary to confirm the proposed ratios, as pointed out by the authors, so multiple variables can be combined, increasing the statistic power. In consequence, the conclusions are still limited, but these findings are promising and worthy of future research.

 The pathological/biochemical mechanism to explain the increase of S100B and NSE in sTBI has been described and makes perfect sense. The protein S100B is produced and released primarily by astrocytes in the central nervous system and is a peripheral biochemical marker of neural injury, including reactive gliosis, astrocytic death, and/or blood-brain barrier dysfunction. Neuron-specific enolase (NSE) is a glycolytic pathway enzyme, predominantly neuronal, so increased CSF/serum levels of NSE and GFAP indicate neuronal and astrocyte damage.

 We are still puzzled by the role of hK6 and PGDS and further research should focus also on describing the underlying cellular mechanisms related to the presented findings.

 It is also noticeable that S100B and NSE proteins are also expressed in other non-neural cell types under physiological and pathological conditions, so future studies should control for that, especially in trauma.

I confirm that I have read this submission and believe that I have an appropriate level of expertise to confirm that it is of an acceptable scientific standard.

# Peer review: Reviewer response for version 1

**DOI:** 10.5256/f1000research.22476.r56390

**Published:** 2019-11-12

**Authors:** Firas H. Kobeissy

**Affiliations:** 1 Department of Biochemistry and Molecular Genetics, Faculty of Medicine, American University of Beirut, Beirut, Lebanon

We read with interest the study by Chen *et al.*, where the authors have assessed (hK6)  and (PGDS) as new markers of injury indicators coupled with NSE and S100B. 

 These two proteins [(hK6)  and (PGDS)] are of high interest and worth looking at; however, this study lacks enough power (n=10 exp vs 10 control) which is not enough to draw any kind of discriminatory level.

 N of patients should be increased and other demographic data should be included as well. The TBI patients assessed are in the severe level and as the authors have mentioned that both UCH-L1 and GFAP have been considered the gold standard by the FDA specifically for severe TBI which is the case in this study.

 This is in contrast to the S100B and NSE which have been criticized for their non-specificity (muscle and red blood cells derived!). Thus, I think these data should be contrasted to UCH-L1 and GFAP with higher N.

 It is of interest as to why the CT scan was not included in the comparison.

I confirm that I have read this submission and believe that I have an appropriate level of expertise to state that I do not consider it to be of an acceptable scientific standard, for reasons outlined above.

# Peer review: Reviewer response for version 1

**DOI:** 10.5256/f1000research.22476.r54418

**Published:** 2019-10-18

**Authors:** Joško Osredkar

**Affiliations:** 1 Institute of Clinical Chemistry and Biochemistry, University Medical Centre Ljubljana, Ljubljana, Slovenia

Time for blood sampling after the brain injury is critical, especially for S100B. Biomarker S100B is increased within minutes after blood-brain-barrier (BBB) disruption. The passage to the systemic circulation is critical, especially the fact that only the BBB is damaged and not the brain. Another point is that most of these markers are expressed to varying degrees outside the central nervous system.

 If possible a more precise time between injury and sampling would be an additional help in the interpretation. In the next study, send a sample within the specified time to calculate the difference in S100B concentrations.

 In severe traumatic brain injury, we have cerebrospinal fluid (CSF) as well and it would be helpful to determine albumin (in CSF and plasma) to see if there is a brain or vessel damage.

I confirm that I have read this submission and believe that I have an appropriate level of expertise to confirm that it is of an acceptable scientific standard.
